# Poly(chitosan-ester-ether-urethane) Hydrogels as Highly Controlled Genistein Release Systems

**DOI:** 10.3390/ijms22073339

**Published:** 2021-03-24

**Authors:** Martyna Zagórska-Dziok, Patrycja Kleczkowska, Ewa Olędzka, Ramona Figat, Marcin Sobczak

**Affiliations:** 1 Department of Technology of Cosmetic and Pharmaceutical Products, Faculty of Medicine, University of Information Technology and Management in Rzeszow, 2 Sucharskiego St., 35-225 Rzeszow, Poland; mzagorska@wsiz.edu.pl; 2 Centre for Preclinical Research (CBP), Department of Pharmacodynamics, Medical University of Warsaw, 1B Banacha St., 02-097 Warsaw, Poland; patrycja.kleczkowska@wum.edu.pl; 3 Military Institute of Hygiene and Epidemiology, 4 Kozielska St., 01-163 Warsaw, Poland; 4 Chair of Analytical Chemistry and Biomaterials, Department of Biomaterials Chemistry, Faculty of Pharmacy, Medical University of Warsaw,1 Banacha St., 02-097 Warsaw, Poland; eoledzka@wum.edu.pl; 5 Department of Environmental Health Sciences, Faculty of Pharmacy, Medical University of Warsaw,1 Banacha St., 02-097 Warsaw, Poland; rfigat@wum.edu.pl

**Keywords:** active substance delivery systems, biomedical hydrogels, active substance-controlled release, genistein, hydrogels for cosmetology, hydrogels for dermatology, transdermal active substance delivery systems

## Abstract

Polymeric hydrogels play an increasingly important role in medicine, pharmacy and cosmetology. They appear to be one of the most promising groups of biomaterials due to their favorable physicochemical properties and biocompatibility. The objective of the presented study was to synthesize new poly(chitosan-ester-ether-urethane) hydrogels and to study the kinetic release of genistein (GEN) from these biomaterials. In view of the above, six non-toxic hydrogels were synthesized via the Ring-Opening Polymerization (ROP) and polyaddition processes. The poly(ester-ether) components of the hydrogels have been produced in the presence of the enzyme as a biocatalyst. In some cases, the in vitro release rate of GEN from the obtained hydrogels was characterized by near-zero-order kinetics, without “burst release” and with non-Fickian transport. It is important to note that developed hydrogels have been shown to possess the desired safety profile due to lack of cytotoxicity to skin cells (keratinocytes and fibroblasts). Taking into account the non-toxicity of hydrogels and the relatively highly controlled release profile of GEN, these results may provide fresh insight into polymeric hydrogels as an effective dermatological and/or cosmetological tool.

## 1. Introduction

Hydrogels are a group of biomaterials that are perceived as a valuable tool in both cosmetology and dermatology, as they are efficient carriers of various therapeutic substances used in the treatment of a wide range of skin diseases [1,2]. This is mainly due to their ease of use and minimal range of possible side effects that are often observed in oral or intravenous drug administration. The multitude of hydrogel matrices currently under development enables many active substances, both hydrophilic and hydrophobic, to be incorporated into their structures [3]. This can be achieved through the formation of inclusion complexes, the production of nanoparticles, liposomes, microspheres and/or micelles [1,3,4,5,6]. Hydrogels based on natural and synthetic polymers, owing to their high biocompatibility, non-toxicity, biofunctionality, biodegradability, relatively low immunogenicity as well physical properties similar to natural tissues, are used for materials with many biomedical applications [1], namely, for the production of contact lenses, artificial organs and materials for the reconstruction and regeneration of cartilage, for tissue engineering and reconstructive surgery, as dressings for the healing of wounds as well as release systems for various compounds with therapeutic effects [1,7,8,9,10,11,12,13]. In recent years, hydrogel biomaterials that are characterized by static properties or play the role of “smart” hydrogels that can respond to different types of stimuli have been more and more dynamically developed [14]. Furthermore, there is also an increasing interest in this type of material in the treatment of various dermatological diseases and beauty deficiencies. Indeed, the use of hydrogels as carriers of medicinal agents, both local and systemic, may prove to be an effective tool against skin diseases, which has been indicated by numerous scientific reports [15,16,17].

A number of forms of hydrogels containing numerous pharmacologically active substances in their structures and intended for use in the treatment of numerous skin diseases have been developed. These biomaterials also have a positive effect on the wound healing process and are used as fillers in aesthetic medicine treatments and as scaffolds for tissue regeneration [18,19,20,21,22,23,24,25,26]. Genistein (GEN) is an isoflavonoid with a wide spectrum of biological activity, which was first isolated from the *Genista tinctoria* L. in 1899, but is also present in many raw materials of plant origin [27]. Being a phytoestrogen makes it interesting as a potent valuable material for cosmetology as well as dermatology [28]. Estrogen receptors are found in various areas of the skin, where they play a specific, well-defined role. The similarity of the chemical structure of GEN to estrogens allows it to bind to estrogen receptors located in the cell nucleus, which results in a change in the expression of many genes that play an important role in many physiological and metabolic processes of the body [29]. Based on the current scientific reports, it can be concluded that the incorporation of GEN into the structure of hydrogels may prove helpful in the context of dermatology and cosmetology, because this phytoestrogen may contribute to the inhibition of skin aging processes [30,31], reduce the appearance of brown “age spots” on the body and improve the elasticity and firmness of our skin [32,33]. Additionally, the possibility of inhibiting the multiplication of many microorganisms by GEN indicates that this compound may also prove to be a useful tool in the fight against various bacterial and fungal diseases of the skin [34,35,36,37].

Many research groups have also attempted to evaluate the effects of GEN by conducting both in vitro and in vivo analysis. The obtained results indicate that in vitro GEN can inhibit the excessive growth of fibroblasts, which prevents their excessive proliferation and the formation of unsightly scars [38]. Research also indicates that GEN may counteract the inhibition of collagen biosynthesis by fibroblasts and protect skin cells from the harmful effects of UV radiation [39,40]. In addition, this compound reduces inflammation, improves the wound healing process, reduces oxidative and photodynamic damage and promotes DNA repair processes [32,41,42]. Research on skin ageing has also shown that GEN can inhibit this process by increasing the thickness of collagen fibers, increasing the resistance of the skin to breakage and reducing the levels of TGF-b1, MMP-2, MMP-9, VEGF, TIMP-1 and TIMP-2 [43,44]. Furthermore, the clinical trials showed that isoflavones, including GEN, have a positive effect on the proliferation of epidermal cells, the production of collagen, the reduction of fine wrinkles and the improvement of skin elasticity [41].

Even though GEN has skin protecting activity, its sustained effect is still limited due to lower stability in cosmeceutical products [45]. In order to enhance stability and prolong activity, delivery technologies have greater impact in cosmeceutical sectors. For example, GEN-loaded nanoemulsions were prepared with 250 nm sized nanoparticles and enhanced delivery of isoflavones to the skin with higher skin protecting activity [46]. In another study, nanosized liposome-encapsulated GEN in nanoparticles the size of about 80 nm was tested on rat skin, with or without hair [47]. In addition to the nanosized liposomes, the hairs on the skin also affect the delivery of the GEN compound to the skin [45]. No commercial hydrogels containing genistein dedicated to cosmetology and dermatology have been yet developed. This is probably due to the unsatisfactory control of the kinetics of genistein release from such carriers. However, there is still intensive work on this type of materials in many scientific and industrial centers.

Given the above, in this work, we have shown for the first time new synthesized poly(chitosan-ester-ether-urethane) hydrogels characterized by highly GEN controlled release. A significant novelty of our findings relies on using biodegradable hydrogels with different structures as an efficient solution for the modification of GEN-release properties. It is important to mention that the obtained hydrogels possess a desired safety profile in terms of lack of cytotoxicity towards skin cells. We believe that such obtained hydrogels could be practically applied for dermatology and cosmetology.

## 2. Results and Discussion

### 2.1. Synthesis of CL, LA and PEG Copolymers

The main aim of the present work was the synthesis and characterization of poly(chitosan-ester-ether-urethane) hydrogels as new and potential GEN carriers. Our main intention was to establish a relationship between the composition and physicochemical properties of the synthesized hydrogels and the amount of GEN released from them. As a consequence, the hydrogels were obtained by a three-step method.

In the first step (1), copolymers *rac*-lactide (*rac*-LA) and poly(ethylene glycol) (PEG) (PLA-PEG) or ε-caprolactone (CL) and PEG (PCL-PEG) were synthesized via enzyme Ring-Opening Polymerization process (e-ROP) (Table 1). The e-ROP process was carried out at 80 °C during 7 days in toluene as a medium. Immobilized lipase B from *Candida antarctica* (CALB) was used as a biocatalyst. The molar ratio of the monomers (*rac*-LA or CL) to PEG was: 20:1, 25:1, 30:1, 35:1 or 40:1. The chemical structure of the obtained copolymers was confirmed by ^13^C, ^1^H NMR and FTIR studies (Experimental section). As shown in Table 1, the synthesized copolymers were characterized by a similar value of Mn (3200–3700 g/mol), but a different content of PEG units in the polymer chain (circa 11, 15 and 20 mol. % PEG). Reaction yields ranged from 69 to 88%.

### 2.2. Swelling and Biodegradation of Hydrogels Studies

**Scheme 1:**
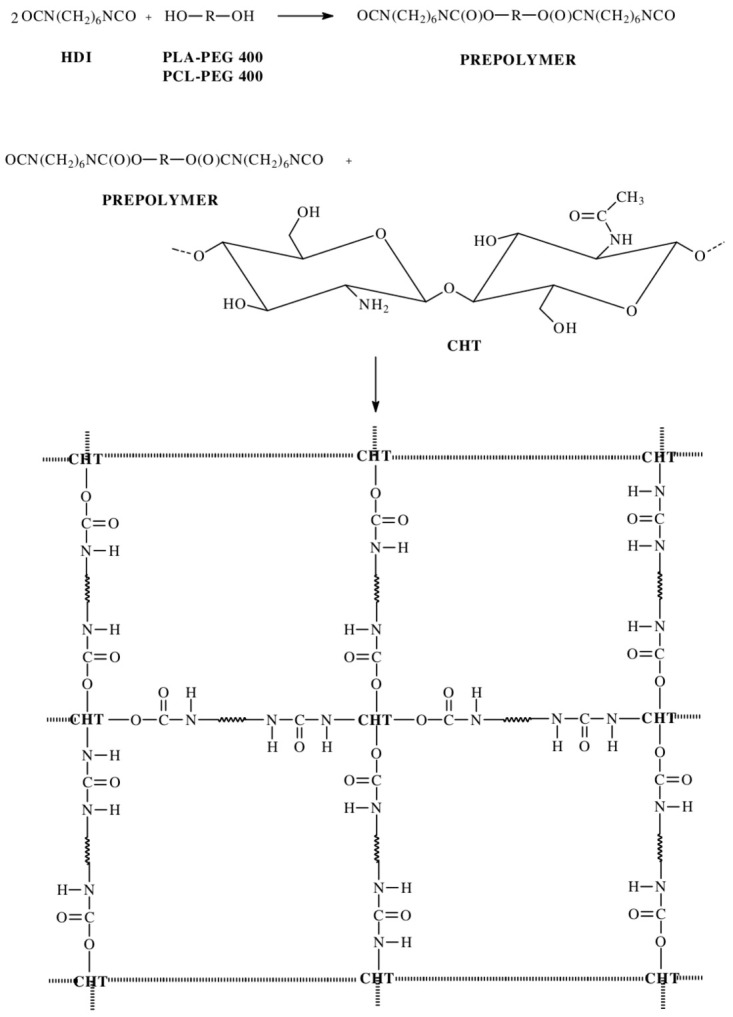
Hydrogel synthesis.

In the next stage of this study, the hydrogels were synthesized using prepolymer methods such as: (2) preparation of prepolymers and (3) polyaddition of prepolymers and chitosan (CHT) (Scheme 1). In step (2), various PLA-PEG or PCL-PEG were reacted with hexamethylene diisocyanate (HDI) in a −NCO/−OH molar ratio of 2.05:1. In step (3), the prepolymer was reacted with CHT in a −NCO(prepolymer)/−OH(or NH_2_) (CHT) ratio of 1.5:1. The dibutyltin dilaurate (DBDLSn) was used as a polyaddition catalyst (Table 2).

The swelling capacity of the obtained hydrogels was determined (Table 2). The value of the coefficient of the mass swelling ratio (MSR) was 375%, 311%, 284%, 297%, 258% and 211% after 4 h for HPUCHT-1, HPUCHT-2, HPUCHT-3, HPUCHT-4, HPUCHT-5 and HPUCHT-6, respectively. As we can easily see, the value of the MSR depended on the nature of the polyester used and the content of the PEG units in the hydrogel chain. As expected, MSR increases with the growth of hydrophilic fragments—PEG units in the polymer chain. 

### 2.3. In Vitro Release Studies of GEN from Hydrogels

**Figure 1:**
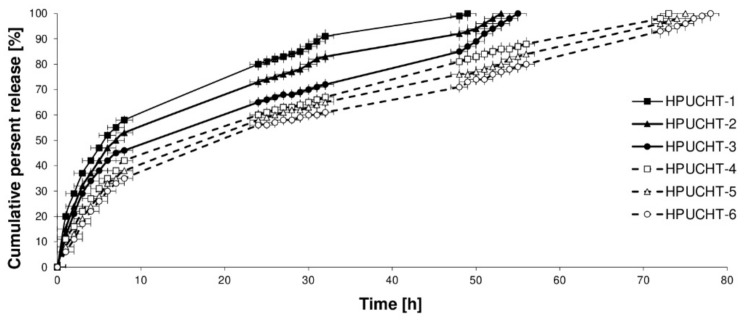
GEN release profiles from the hydrogels.

GEN was loaded into hydrogels using the incorporation method. The mean weight of the devices developed was approximately 200 mg, corresponding to approximately 10 mg of GEN (active substance content was adjusted to about 5%). In vitro studies of the release of GEN from obtained hydrogel materials were determined at pH 7.4 and 37 °C for 80 h (Figure 1). The ordinate of the plot was calculated based on the cumulative amount of GEN released with respect to its initial amount in the hydrogels.

As mentioned above, our intention in this work was to investigate the influence of two factors on the kinetic release of GEN. The first one was the content of the PEG units in the copolymer; the second was the nature and properties of the polymer forming the copolymer (PLA or PCL).

The rate of the GEN release decreased as follows: HPUCHT-1 (obtained from PLA-PEG-1 (80:20)) > HPUCHT-2 (PLA-PEG-2 (85:15)) > HPUCHT-3 (PLA-PEG-3 (88:12)) > HPUCHT-4 (PCL-PEG-1 (80:20)) > HPUCHT-5 (PCL-PEG-2 (84:16)) > HPUCHT-6 (PCL-PEG-3 (89:11)).

The data points obtained for the drug release studies were subject to zero-order and first-order kinetics as well as to the Korsmeyer−Peppas models for the evaluation of the kinetic and mechanism of GEN release from hydrogels (Table 3). As is known from the literature, according to the Korsmeyer−Peppas model, for the diffusion-degradation-controlled drug release system, the release exponent value n is in the range of 0.45 and 0.89 (anomalous, non-Fickian), whereas when n is close to 0.45, the diffusion (Fickian diffusion) predominates in the process and, in the opposite case, n > 0.89, the model corresponds to the super case II transport [48,49].

In our study, it was observed that the kinetic release of GEN is mainly influenced by the content of the PEG units in the copolymers used for the preparation of hydrogels. For example, approximately 80.8%, 73.4% and 65.2% of GEN were released after 24 h from the HPUCHT-1, HPUCHT-2 and HPUCHT-3 samples, respectively. This is relatively little, because 60.7%, 58.3% and 56.8% of GEN were released after 24 h from the HPUCHT-4, HPUCHT-5 and HPUCHT-6 materials composed of PCL segments in different molar ratios (Table 2). Thereby, we can conclude that GEN was released more rapidly from materials created of PLA polymers than from PCL segments. After 49, 53 and 55 h, almost 100% of GEN was released from hydrogels composed of PLA units (Figure 1). In the case of PCL polymer materials, the time for the GEN releasing in 100% was longer and ranged up to 78 h (Figure 1). This is undoubtedly related to the higher hydrophilicity of hydrogels obtained from PLA-PEG copolymers compared with those obtained from PCL-PEG copolymers. The Mn of the PLA-PEG or PCL-PEG copolymers used in the hydrogel synthesis did not appear to affect the amount of GEN released. This could be because the Mn values of all copolymers were similar (3200–3700 g/mol) (which was consistent with the authors’ assumptions). It was also discovered that the higher the PEG content in a given matrix composed of PLA or PCL units, the higher the amount of GEN released.

The GEN release kinetic from hydrogels obtained from PCL-PEG copolymers (HPUCHT-4, HPUCHT-5, HPUCHT-6) followed the near-zero-order model (R^2^ was 0.923, 0.925 and 0.941, respectively). Furthermore, for the HPUCHT-3 sample (hydrogel containing the lowest percentage of PEG units, but composed of PLA units), the GEN release was also close to the zero-order kinetics (R^2^ = 0.921). Furthermore, it was noted that GEN was released from the HPUCHT-1 and HPUCHT-2 samples with rather first-order kinetics (R^2^ was 0.915 and 0.940 (Table 3). The analysis of GEN release data using the Korsmeyer−Peppas model suggested that all the hydrogels were governed rather by a non-Fickian transport (n = 0.452–0.609).

**Figure 2:**
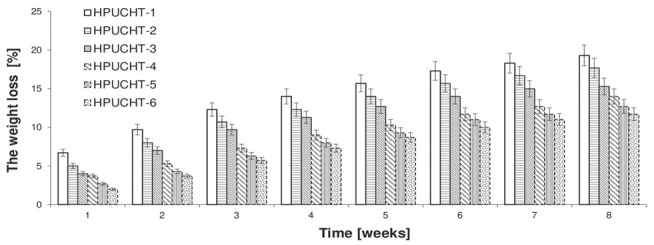
The weight loss (WL) of the obtained hydrogels.

The biodegradation of the blank hydrogels has also been carried out. Hydrolytic degradation test of the resulting carriers was conducted under the same conditions as the GEN release experiments. The degradation process was characterized by the plotting of the weight loss (WL) of hydrogels versus time. The results are shown in Figure 2.

The degradation rate of the hydrogels was found to be similar to that of the GEN release profile (Figure 1). The blank hydrogels degradation rate was as follows: HPUCHT-1 > HPUCHT-2 > HPUCHT-3 > HPUCHT-4 > HPUCHT-5 > HPUCHT-6. Similarly, hydrogels formed of PLA polymer degrade faster than that obtained from PCL segments. Apparently, this corresponds to a higher hydrophilicity of the PLA polymer. In addition, the rate of degradation of the hydrogel has increased with an increase in the content of the PEG units in the copolymer chain. In summary, on the basis of the data obtained from the kinetic release and mathematical models, it can be concluded that in some cases, GEN was released with relatively high control from the synthesized hydrogel materials.

Certain GEN vectors, including nanoparticles, nanocapsules or nanoemulsions, have been developed and thoroughly studied [50,51,52,53,54]. For instance, GEN was released from zein and zein/carboxymethyl CHIT nanoparticles at a rate of about 60% after 24 h. The authors suggest a controlled release, although the “burst release” phenomenon can be observed in the kinetic profiles. Moreover, the authors did not match the obtained release results with any kinetic models [50]. GEN-loaded solid lipid nanoparticles (SLNs) and nanostructured lipid carriers (NLCs) were obtained by Andrade et al. [50]. Skin permeation studies have shown that lipid nanoparticles have increased GEN skin retention but GEN release kinetics have not been fully controlled [50]. GEN-loaded PLA nanocapsules (GEN-NC) were prepared in paper [52] by interfacial deposition of pre-formed polymers (nanoprecipitation). Permeation experiments have demonstrated that a higher amount of GEN reaches deeper layers of the skin and increased penetration was achieved when GEN-NC was incorporated into a semi-solid gel formulation. It indicated that GEN-NC could be a promising nanocarrier system for GEN skin delivery, but this active substance release kinetics was not fully controlled. Nanoemulsions composed of isopropyl myristate/dioleylphosphaditylcholine/oleylamine with GEN have also been obtained [53]. The incorporation of GEN into nanoemulsions significantly increased the retention of this isoflavone in epidermis and dermis. These results were supported by confocal images. Such formulations exhibited antiherpetic activity in vitro against herpes simplex virus 1 and herpes simplex virus 2. The results shows that the GEN-loaded nanoemulsions developed in this study are promising options for herpes treatment. Nanoemulsions containing isoflavone aglycone-rich fraction (NE IAF) and derivative semi-solid hydrogels composed of hyaluronic acid have been obtained at work [54]. NE IAF containing GEN has been prepared and, in some formulations, hyaluronic acid has been added to obtain hydrogels. The distribution of GEN in skin layers has been evaluated. These results showed the potential of formulations for topical skin applications. However, the kinetics of GEN release from the above-mentioned carriers have not been investigated.

In the light of the cited literature examples, we are unfortunately of the opinion that the comparison of these biomaterials with the hydrogels produced in our work is quite difficult due to the fact that our materials have been synthesized from a different type of polymer and thus have a different structure. In our view, the developed biodegradable poly(chitosan-ester-ether-urethane) hydrogels (HPUCHT-3, HPUCHT-4, HPUCHT-5, HPUCHT-6) are characterized by relatively high release control and may constitute a potential material for further dermatological applications.

### 2.4. Genotoxic Test

The umu-test was used to evaluate the genotoxicity of the blank hydrogels (Table 4). In the umu-test, it was found that none of the samples tested were toxic to *S. typhimurium* (G > 0.5) (Table 4). None of the tested extracts exhibited a genotoxic effect (IR < 1.5) with or without metabolic activation (IR < 1.5).

### 2.5. Cytotoxicity Assessment

#### 2.5.1. Neutral Red Uptake Assay

**Figure 3:**
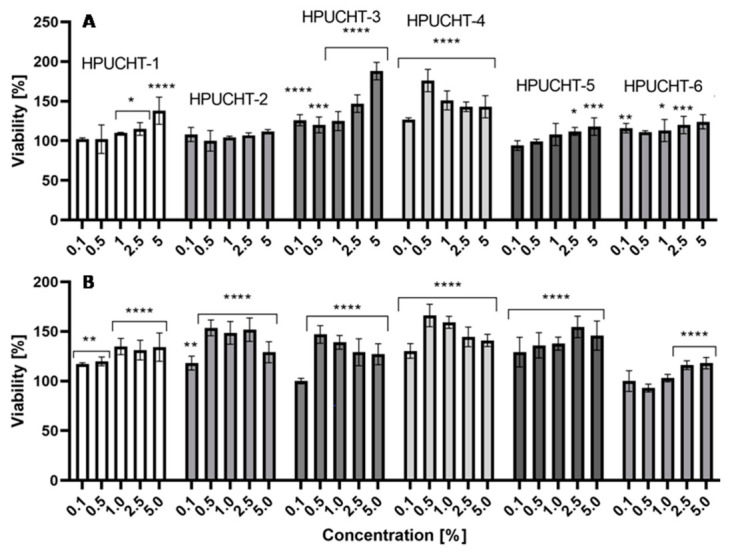
Effect of different concentrations of extracts (0.1–5%) from hydrogels on NR uptake in cultured fibroblasts after 2 h (**A**) and 24 h (**B**) of exposure. **** *p* ≤ 0.0001, *** *p* ≤ 0.001, ** *p* ≤ 0.01, * *p* ≤ 0.05 versus the control (100%).

**Figure 4:**
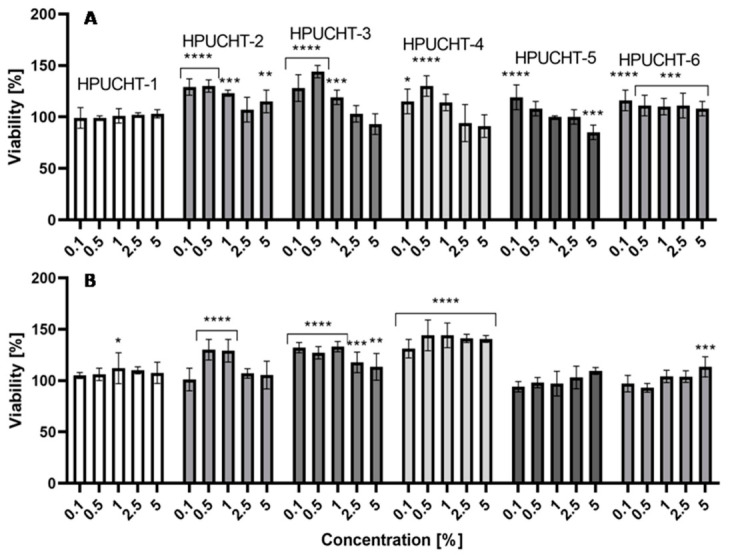
Effectof different concentrations of extracts (0.1–5%) from hydrogels on NR uptake in cultured HaCaT cells after 2 h (**A**) and 24 h (**B**) of exposure. **** *p* ≤ 0.0001, *** *p* ≤ 0.001, ** *p* ≤ 0.01, * *p* ≤ 0.05 versus the control (100%).

In order to assess the biocompatibility of the obtained hydrogels and the possibility of their use in the treatment of various skin diseases, cytotoxicity tests were carried out on skin cells located in different layers of the skin—fibroblasts and keratinocytes. For this purpose, these cells were exposed to the tested hydrogels for 2 and 24 h. The cell viability was estimated using the Neutral Red (NR) and Alamar Blue (AB) assays. The obtained results revealed that the hydrogels tested were characterized by high biocompatibility, as no cytotoxic effects were observed in any of the hydrogels. The analysis conducted showed that the effect of the experiments on keratinocytes (HaCaT) and fibroblasts (BJ) varies depending on the type of hydrogel tested and the time of exposure to hydrogel extracts. The NR assay performed on fibroblasts showed that the extended exposure time to the hydrogels tested had a positive effect on the viability of these cells, which is probably related to the release of GEN from hydrogels and its positive effect on the skin cells of interest. Notably, promising results for HPUCHT-3 and HPUCHT-4 hydrogels were observed after a shorter exposure time. An average cell viability of 188% was recorded for HPUCHT-3 hydrogel at the highest extract concentration (5%). After longer exposure, all the hydrogels tested were found to stimulate an increase in the viability of the fibroblasts. However, in the case of HPUCHT-6 hydrogel, this effect was observed only at the two highest extract concentrations (2.5 and 5.0%) (Figure 3A,B). In the case of keratinocytes, such a significant effect of the prolonged exposure time on the increase in the viability of these cells was unnoted (Figure 4A,B). Increased viability of keratinocytes treated with hydrogels tested was also reported to be lower compared to fibroblasts. The most promising results have, indeed, been achieved for HPUCHT-2, HPUCHT-3 and HPUCHT-4 hydrogels. On the other hand, the remaining hydrogels did not have a positive effect on the viability of HaCaT cells as a response to the extended exposure time, but still had no cytotoxic activity (Figure 4B).

#### 2.5.2. Alamar Blue Assay

**Figure 5:**
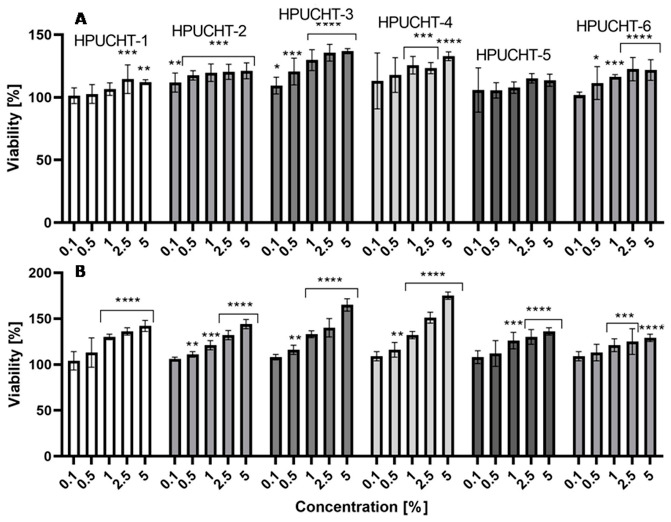
Thereduction of resazurin after 2 h (**A**) and 24 h (**B**) of exposure to extracts (0.1–5%) from hydrogels in cultured fibroblasts. **** *p* ≤ 0.0001, *** *p* ≤ 0.001, ** *p* ≤ 0.01, * *p* ≤ 0.05 versus the control (100%).

In the case of the Alamar Blue assay, no cytotoxicity of the analyzed hydrogels was observed for eithertypes of skin cells. These analyses showed that the longer exposure time (up to 24 h) to the tested hydrogels significantly increased the metabolic activity of the fibroblasts, which reached 165 and 175% for HPUCHT-3 and HPUCHT-4 hydrogels at the highest concentrations (Figure 5A,B). On the other hand, better results were obtained for a shorter incubation time for keratinocytes, indicating a higher sensitivity of these cells to the action of the hydrogels tested.

**Figure 6:**
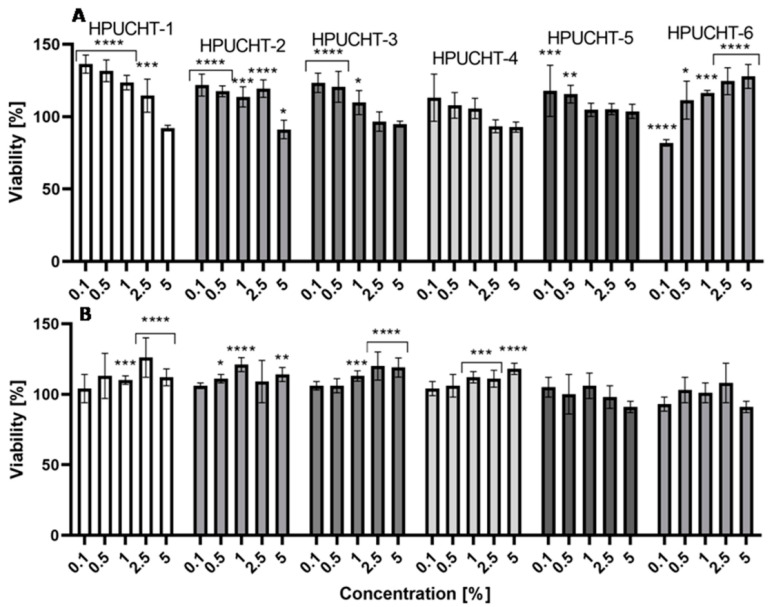
Thereduction of resazurin after 2 h (**A**) and 24 h (**B**) of exposure to extracts (0.1–5%) from hydrogels in cultured HaCaT cells. **** *p* ≤ 0.0001, *** *p* ≤ 0.001, ** *p* ≤ 0.01, * *p* ≤ 0.05 versus the control (100%).

It should be noted, however, that no cytotoxic effect was observed after 2 and 24 h of exposure of keratinocytes to the hydrogels tested and most of the hydrogels stimulated the metabolic activity and viability of these cells (Figure 6A,B). The studies carried out, thus, show a lack of in vitro cytotoxicity of the hydrogels tested, which gives hope for the possibility of their potential application after appropriate in vivo analyses and clinical trials.

**Figure 7:**
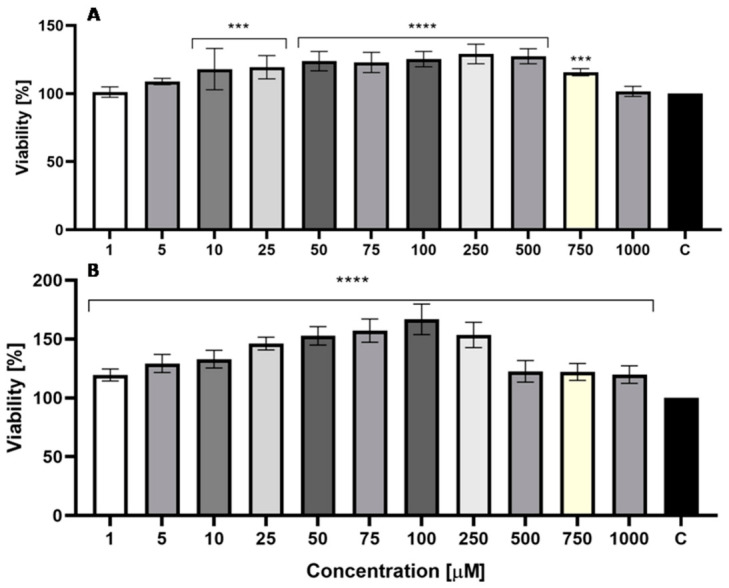
The reduction of resazurin after 2 h (**A**) and 24 h (**B**) of exposure to GEN (1–1000 µM) in cultured fibroblasts. **** *p* ≤ 0.0001, *** *p* ≤ 0.001 versus the control (100%).

**Figure 8:**
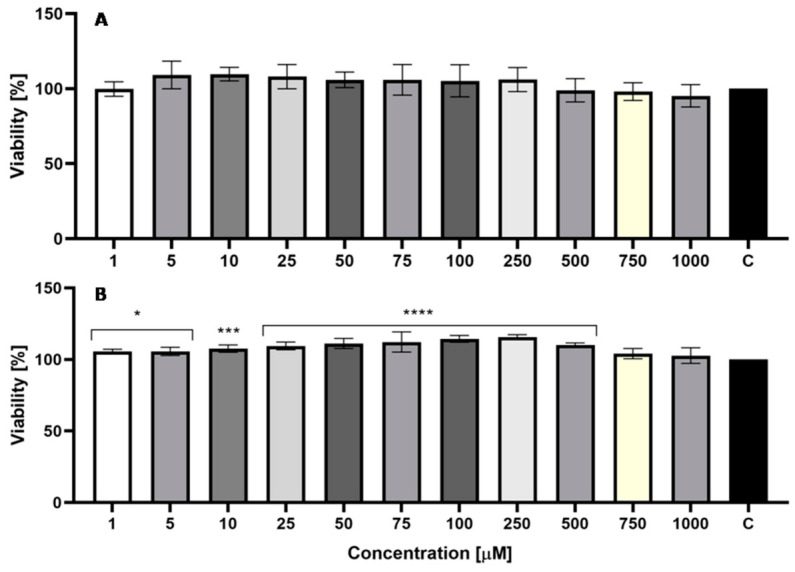
The reduction of resazurin after 2 h (**A**) and 24 h (**B**) of exposure to GEN (1–1000 µM) in cultured HaCaT cells. **** *p* ≤ 0.0001, *** *p* ≤0.001,* *p* ≤ 0.05 versus the control (100%).

As part of the studies, the effect of GEN on the cell lines tested was also assessed. The obtained results indicate that this compound has no negative effect on both keratinocytes and fibroblasts in the tested concentration range (1–1000 µM). In the case of fibroblasts, it was observed that a 2-h incubation of these cells with GEN causes a statistically significant increase in the metabolic activity of these cells (the concentrations was ranged from 100 to 750 µM), and after 24-h incubation with 100 µM of GEN, the increase in this activity was almost 70% compared to the control cells (untreated with GEN). Concentrations of 250–1000 μM also significantly increased the proliferation of fibroblasts. However, as the concentration of GEN increased, this effect was weaker, which could indicate a cytotoxic effect of GEN at higher concentrations (above 1000 μM) (Figure 7A,B). In the case of keratinocytes, no statistically significant increase in cell proliferation was observed after 2-h incubation, while the longer incubation period resulted in an increase in the metabolic activity of these cells exposed to GEN at concentrations of 1–500 μM. Higher concentrations (750 and 1000 μM) did not cause a decrease in cell proliferation compared to control cells, but, as in the case of fibroblasts, a downward trend in the activity of these cells was observed along with an increase in the GEN concentration (Figure 8A,B).

The increase in cell viability and the rate of proliferation following the use of GEN may be due to an improvement in the mitochondrial membrane potential and reduced release of reactive oxygen species by increasing the glutathione (GSH) level, as indicated by other authors [33]. The use of hydrogel matrices directly to the skin may also prove to be an effective route of administration of this isoflavone, as GEN has low bioavailability and is rapidly metabolized following oral administration, which has a significant effect on the absorption of this compound [55,56]. This is supported by the results obtained by Huang et al., which demonstrate that topical administration may be an effective route for the delivery of soy isoflavones, including GEN [57]. Consequently, the possibility of controlled release of GEN from the biodegradable hydrogels developed in this study, with the simultaneous lack of their cytotoxic effect on skin cells, may suggest the possibility of their potential use in the treatment of dermatological diseases after more detailed in vivo and clinical studies. Taking into account the noticeable lack of effective systems for controlled release of GEN, which has an extremely beneficial effect on the skin described in the introduction to this work, the hydrogels developed may be an opportunity to improve the skin condition of many people currently struggling with common skin diseases.

## 3. Materials and Methods

### 3.1. Materials

1,6-diisocyanatohexane (hexamethylene diisocyanate, HDI, 98%, Aldrich, Poznan, Poland), 3,6 dimethyl 1,4 dioxane 2,5 dione (*rac*-lactide, *rac*-LA, 99%, Sigma-Aldrich, Poznan, Poland), acetic acid (CH_3_COOH, ≥99%, Sigma-Aldrich, Poznan, Poland), antibiotics (Penicillin-Streptomycin, Life Technologies, Bleiswijk, The Netherlands), ε Caprolactone, (2 Oxepanone, CL, 99%, Aldrich, Poznan, Poland), chitosan (CHT, low molecular weight, 75% deacetylated), dibutyltin dilaurate (DBDLSn, >96%, Sigma-Aldrich, Poznan, Poland), dichloromethane (DCM, CH_2_Cl_2_, ≥99.8%, POCh, Gliwice, Poland), DMEM (Dulbecco’s Modification of Eagle’s Medium, Biological Industries, Beit Haemek, Israel), ethyl alcohol (ethanol, C_2_H_5_OH, 96%, Sigma-Aldrich, Poznan, Poland), FBS (Fetal Bovine Serum, Biological Industries, Genos, Lodz, Poland), genistein (GEN, >98%, Tokyo Chemical Industry Co. LTD., Tokyo, Japan), hydrochloric acid (HCl, ChemPur, Piekary Śląskie, Poland), immobilized lipase B from *Candida antarctica* (CALB) (Sigma-Aldrich, Poznan, Poland), Neutral Red Solution (NR, 0.33%, Sigma-Aldrich, Poznan, Poland), *N*,*N*-dimethylformamide (DMF, anhydrous, 99.8%, Sigma-Aldrich, Poznan, Poland), phosphate buffered saline (PBS, pH 7.00 ± 0.05, ChemPur, Piekary Ślaskie, Poland), poly(ethylene glycol) 400 (PEG 400, Sigma-Aldrich, Poznan, Poland), resazurin sodium salt (RES, Sigma-Aldrich, Poznan, Poland), toluene (99.8%, POCh, Gliwice, Poland) and trypsin-EDTA solution (Sigma-Aldrich, Poznan, Poland) were used as received.

### 3.2. Synthesis of CL, LA and PEG Copolymers

The polymerization reactions were carried out according to our previously described method with some modifications [58,59,60]. Before the reaction, monomers (CL or rac-LA), PEG and CALB were dried under vacuum at room temperature for 3 h. Next, 0.04 mol CL (or 0.04 mol *rac*-LA) was placed in a three-neck flask equipped with a stirrer and thermometer (under argon atmosphere) and 20 mL of toluene was added. The mixture was stirred at 60 °C for 2 h. Next, an appropriate amount of PEG 400 and CALB (400 mg) was added to the mixture (Table 1). Stirring was continued at 60 °C for 24 h under argon atmosphere. After this time, the enzyme was filtered off. Toluene was removed by evaporation under reduced pressure at room temperature. Next, the cooled product was dissolved in DCM and extracted within cold methanol and distilled water.

### 3.3. Spectroscopy Data

The ^1^H NMR spectrum of *rac*-LA and PEG copolymer (PLA-PEG): 1.57 ppm (-O(O)C-CH(CH_3_)-), 3.63 ppm (O CH_2_-CH_2_-O-), 4.29 ppm (-O(O)C-CH(CH_3_)-OH end group) and 5.17 ppm (-O(O)C-CH(CH3)-).

The ^13^C NMR spectrum of PLA-PEG: 16.7 ppm (-O(O)C-CH(CH_3_)-), 69.0 ppm (-O(O)C-CH(CH_3_)-), 70.5 ppm (-O-CH_2_-CH_2_-O) and 169.3 ppm (-O(O)C-CH(CH_3_)-).

The FTIR spectrum of PLA-PEG (KBr, cm^−1^): 2997 (υ_as_CH_3_), 2947 (υ_s_CH_3_), 2882 (υCH), 1760 (υC=O), 1452 (δ_as_CH3), 1348–1388 (δ_s_CH3), 1368-1360 (δ_1_CH+δsCH3), 1315-1300 (δ_2_CH), 1270 (δCH + υCOC), 1215-1185 (υ_as_COC + r_as_CH3), 1130 (r_as_CH3), 1100-1090 (υ_s_COC), 1045 (υC-CH_3_), 960-950 (rCH_3_ + υCC), 875-860 (υC-COO), 760-740 (δC=0), 715-695 (γC=O), 515 (δ1C-CH_3_ + δCCO), 415 (δCCO), 350 (δ_2_C-CH_3_ + δCOC), 300-295 (δCOC + δ_2_C-CH3), 240 (τCC).

The ^1^H NMR spectrum of CL and PEG copolymer (PCL-PEG): 1.38 ppm (O CH_2_-CH_2_-CH_2_-CH_2_-CH_2_-),1.65 ppm (-O-CH_2_-CH_2_-CH_2_-CH_2_-CH_2_-), 2.31 ppm (-O(O)C-CH_2_-CH_2_-CH_2_-CH_2_-CH_2_-), 3.65 ppm (-O-CH_2_-CH_2_-O-) from PEG mers and (CH_2_ CH_2_-CH_2_-CH_2_-CH_2_-OH end groups), 4.06 ppm (-O-CH_2_-CH_2_-CH_2_-CH_2_-CH_2_-) and 4.23 ppm (-O-CH_2_-CH_2_-O(O)C-CH_2_-CH_2_-CH_2_-CH_2_-CH_2_-).

The ^13^C NMR spectrum of PCL-PEG: 24.7 ppm (-O(O)C-CH_2_-CH_2_-CH_2_-CH_2_-CH_2_-), 25.6 ppm (-O(O)C-CH_2_-CH_2_-CH_2_-CH_2_-CH_2_) 28.4 ppm (-O-CH_2_-CH_2_-CH_2_-CH_2_-CH_2_-), 34.2 ppm(-O(O)C-CH_2_-CH_2_-CH_2_-CH_2_-CH_2_-), 64.2 ppm (-O-CH_2_-CH_2_-CH_2_-CH_2_-CH_2_-), 70.6 ppm(-O-CH_2_-CH_2_-O-) and 173.6 ppm (-O(O)C-CH_2_-CH_2_-CH_2_-CH_2_-CH_2_-).

The FTIR spectrum of PCL-PEG (KBr, cm^−1^): 2944 (ν_as_CH_2_), 1722 (νC=O), 1244 (νC-O).

### 3.4. Hydrogels’ Preparation

The hydrogels were obtained according to the described prepolymer method with some modifications [61]. The prepolymers were obtained through a polyaddition reaction between HDI and PLA-PEG (or PCL-PEG) in a −NCO/−OH molar ratio of 2.05:1, using 2 drops of 0.1 wt% DBDLSn solution in toluene as catalyst. The reactions were performed at 80 °C for 3 h under argon atmosphere to form an isocyanate terminated prepolymer. Next, the dispersion of CHT into glacial acetic acid/DMF mixture (30 mL) in a volume ratio of 50/50 has been prepared. Next, the obtained prepolymer was added to the dispersion of CHT. The reactions were carried out in a −NCO(prepolymer)/−OH (or NH_2_) (CHT) molar ratio of 1.5:1 at 80 °C for 4 h under argon atmosphere. The reaction mixture was then transferred to the distilled water. Precipitated products were separated by filtration and washed with DMF, methanol and acetone. The final products were dried under vacuum for one week. The developed hydrogels were denoted as: HPUCHT-1 (formed from PLA-PEG-1, 80:20), HPUCHT-2 (formed from PLA-PEG-2, 85:15), HPUCHT-3 (formed from PLA-PEG-3, 88:12), HPUCHT-4 (formed from PCL-PEG-1, 80:20), HPUCHT-5 (formed from PCL-PEG-2, 84:16) and HPUCHT-3 (formed from PCL-PEG-3, 89:11).

### 3.5. Swelling and Biodegradation of Hydrogels Studies

The mass swelling ratio (MSR) of hydrogels was determined at 37 °C during 80 h of incubation in PBS. Samples (approximately 0.5 g) in triplicate were submerged in a PBS solution (10 mL) for a given time, and their weights were taken after removing the excessive surface water. The mass swelling ratio was calculated using the following formula:

MSR = ((W2 − W1) / W1) × 100%

where:

W1 is the weight of the initial hydrogel;

W2 is the weight of the swollen hydrogel.

In order to evaluate the percentage of degradation, the hydrogel samples were immersed in PBS at 37 °C for 4 weeks; most importantly, the medium was replaced with fresh PBS every one week. At the end of the experiment, the samples were dried in a vacuum for 48 h. The degree of degradation of hydrogels (in triplicate) was determined by the weight loss (WL) of the samples according to the equation:

WL = [(W1 − W2) / W1] × 100%

where:

WL is the weight loss;

W1 is the weight of dry sample before degradation;

W2 is the weight of the dry sample after degradation.

### 3.6. In Vitro Release Studies of GEN from Hydrogels

GEN was loaded to the hydrogel matrices by physical mixing due to the following procedure. A total of 5.0 % (m/m) of GEN in distilled water/Tween 80 mixture (2% (*w*/*v*) was added to six hydrogel samples (HPUCHT-1, HPUCHT-2, HPUCHT-3, HPUCHT-4, HPUCHT-5 and HPUCHT-6). The hydrogels were left sealed for 24 h. The mixtures were dried under vacuum at room temperature to obtain a GEN-loaded hydrogel films. The in vitro release of GEN from the hydrogels was performed in a PBS buffer (pH 7.4 ± 0.05) containing 2% (*w*/*v*) Tween 80 at 37 °C under stirring. Vials containing hydrogel films were filled with 5.0 mL of PBS buffer (pH 7.4 ± 0.05), sealed and left in 37 °C for 2 h. The solutions were then removed for further testing and replaced by fresh PBS. Subsequent samples were collected at selected intervals. GEN concentration in the in vitro samples was also determined by HPLC (detected at the wavelength of 262 nm). The mobile phase was composed of 70% methanol and 30% water with 0.1% phosphoric acid [62,63]. The release data points were subjected to zero-order, first-order kinetics and Korsmeyer–Peppas models, respectively. Calculations were made on the basis of formulas mentioned below:

Zero-order model:

F=kt

First-order model:

logF = log F_0_ − kt/2.303

Korsmeyer–Peppas model:

F=kt^n^ (F<0.6)

where:

F is the fraction of GEN released up to time (t);

F_0_ is the initial concentration of GEN;

k is the constant of the mathematical models;

n is the exponent of the Korsmeyer–Peppasmodel [48,49].

### 3.7. Measurements

The structures of synthesized materials were characterized using ^1^H and ^13^C NMR techniques (Varian 300 MHz, Palo Alto, CA, USA). The spectra were registered in CDCl_3_. The 1H spectra were recorded under following conditions: 300 MHz, 1 s repetition time, 2 s acquisition time, 32, 64 or 128 scans. The ^13^C spectra: 75.4 MHz, 5 s repetition time, 1.4 s acquisition time, 20,000 scans per each spectrum. Percentage molar value of PEG in a copolymer chain were estimated using the following formulas [64]:

$$\%\;CL=\frac{\frac{I_{PEG}}{2}}{I_{\varepsilon }+\frac{I_{PEG}}{2}}\cdot 100\%$$

$$\%\;LA=\frac{\frac{I_{PEG}}{4}}{I_{\alpha }+\frac{I_{PEG}}{4}}\cdot 100$$

The FTIR spectra of PLA-PEG and PCL-PEG prepolymers were measured from KBr pellets (PerkinElmer spectrometer, Great Britain). The average molecular weight and molecular weight distribution were determined on the LabAllianceGel Permeation Chromatograph equipped with Jordi Gel DVB mixed bed (250 × 10 mm) column and refractive detector, using chloroform as solvent. The flow rate was 1 mL/min. The average molecular weights were calibrated with polystyrene standards.

### 3.8. Genotoxic Test

The umu-test is a bioassay to evaluate the genotoxic potential of environmental samples and chemical compounds. The test detects the induction of the SOS system to the strain *Salmonella typhimurium* TA1535/pSK1002. The SOS system is the bacterial response to the DNA-damaging agents. The test strain is genetically modified- the *umuC* gene activity is linked to the synthesis of β-galactosidase, while other DNA regions responsible for the synthesis of this enzyme were deleted. Therefore, β-galactosidase activity strictly depends on the SOS system induction level and the genotoxic activity of the tested sample [65]. Additionally, the bacteria growth (G) is evaluated by a measurement of an optical density to determine the cytotoxicity of tested samples. The genotoxic potential of the sample was presented as the Induction Ratio (IR)-the β-galactosidase activity of the tested sample relative to the negative control. Samples with IR ≥ 1.5 are considered as genotoxic.

In the present study, the umu-test was carried out in the micro-plate variant according to the ISO guideline, with and without metabolic activation (S9 liver fraction) [66]. Deionized sterile water was used as a negative control, 2-aminoanthracene and 4-nitroquinoline N-oxide were used as positive controls, and phosphate buffered saline (PBS from Gibco, Thermo Fisher Scientific, Darmstadt, Germany) as solvent control. All tested samples were incubated in PBS—100 mg/mL for 24 h, at 37 °C, with shaking. Before the assay, all extracts were sterilized by filtration (0.20 µm). All samples were tested in two-fold dilution series (four concentrations in three replicates; the highest concentration of 66.6 mg/mL).

### 3.9. Cell Culture and Preparation of Hydrogel Extracts

Two human skin cell lines were used in the study: HaCaT cells (normal human keratinocytes; CLS Cell Lines Service, Eppelheim, Germany) and BJ cells (fibroblasts, ATCC^®^CRL-2522™; the American Type Culture Collection, Manassas, VA, USA). Both cell lines were maintained in DMEM medium supplemented with L-glutamine, 4.5 g/L glucose and sodium pyruvate. In order to achieve optimal results, the culture medium was supplemented with 10% (*v*/*v*) FBS and 1% (*v*/*v*) antibiotics (100 U/mL penicillin and 1000 µg/mL streptomycin). Cells were cultured in an incubator at 37 °C in a humidified atmosphere of 95% air and 5% carbon dioxide (CO_2_). After the cultured cells (HaCaT and BJ) had reached proper confluence (about 70–80%), the DMEM culture medium was removed from the culture plate (VWR) and the cells were washed twice with sterile PBS. Subsequently, the cell layer was trypsinized with trypsin/EDTA, and the cells were then resuspended in fresh DMEM medium. In the next step, cells were plated in 96-well flat-bottom plates (separate plates for both cell types) and left for 24 h in the incubator. After HaCaT and fibroblasts were attached to the bottom of the plates, cells were incubated with various concentrations of extracts (0.1, 0.5, 1.0, 2.5 and 5.0%) obtained from six different hydrogels (HPUCHT-1, HPUCHT-2, HPUCHT-3, HPUCHT-4, HPUCHT-5, HPUCHT-6) loaded with GEN. Extracts were obtained by incubating test hydrogels in DMEM culture medium for 24 h on a rocker shaker, after whichappropriate dilutions of these extracts were prepared in DMEM. Before the assay started, all extracts were sterilized by filtration (0.20 µm). The cells were then treated with the hydrogel extracts for 2 and 24 h in an incubator. Control samples were cells grown in the DMEM medium without the addition of hydrogel extracts.

### 3.10. Cytotoxicity Assays

#### 3.10.1. Neutral Red Uptake Assay

In order to evaluate the cytotoxicity of the tested hydrogels on HaCaT and BJ cells, the Neutral Red Uptake Assay (Sigma Aldrich, Poznan, Poland) was used. This test was performed based on the procedure described previously [67]. After 2 and 24 h of exposure to extracts of the six GEN-loaded hydrogels, cells were incubated for 2 h with a neutral red dye (40 µg/mL), which was dissolved in serum-free DMEM medium. After incubation with NR, cells were washed with sterile PBS and 150 µL of decolorizing buffer (C_2_H_5_OH/CH_3_COOH/H_2_O, 50%/1%/49%) was added to each well to release cellular dye into the PBS solution. After shaking the cells for 15 min the absorbance of the dissolved dye at λ = 540 nm was determined using a FilterMax F5 Multi-Mode microplate reader (Thermo Fisher). The mean absorbance of the control cells (untreated with the extracts) was taken as 100% cell viability and used to calculate the percentage of viable cells in the experimental samples treated with the test hydrogels. As a part of the study, three independent experiments were carried out, in which each concentration of extracts was tested using four replicates.

#### 3.10.2. Alamar Blue Assay

The cytotoxicity assessment of the hydrogels tested on skin cells was also carried out using the Alamar Blue test. The protocol described by Page et al. [68] has been used for this purpose. After 2 and 24 h of exposure of HaCaT and BJ cells (on separate 96-well plates) to individual concentrations of the analyzed hydrogel extracts (0.1 to 5% concentration range), a resazurin solution (with a final concentration of 60 μM) was added to the well and incubated for 2 h at 37 °C in the dark. The fluorescence of the samples was then measured at λ = 570 nm using a microplate reader (FilterMax F5, Thermo Fisher). The experiments were performed in three independent experiments, in which the fluorescence of the cells in four wells was measured for each extract concentration. The results were expressed as a percentage of cell viability compared to the control sample (100%), which were cells grown in DMEM medium without any addition of hydrogel extracts. Additionally, in order to assess the effect of GEN (not loaded into hydrogel structures) on the tested cells, fibroblasts and keratinocytes were incubated with GEN in the concentration range of 1–1000 µM for 2 and 24 h and the cytotoxicity assessment was performed.

### 3.11. Statistical Analysis

Values of different parameters were expressed as the mean ± standard deviation (SD). Two-way analysis of variance (ANOVA) and Bonferroni posttest between groups were performed at the level *p* < 0.05 to evaluate the significant differences between values. Statistical analyses were performed using GraphPad Prism 8.4.3 (GraphPad Software, Inc., San Diego, CA, USA).

## 4. Conclusions

In the present work, new non-toxic and biodegradable hydrogels for controlled release of GEN have been obtained and characterized. The developed materials have been obtained using a three-step method. The poly(ester-ether)s components have been synthesized by an e-ROP process catalyzed by CALB. In the next steps, hydrogels were obtained by a prepolymer method using CHT and HDI, in the presence of DBDLSn as a catalyst. The in vitro release study showed that the release rate of GEN was highly dependent on the composition of newly developed poly(chitosan-ester-ether-urethane) hydrogels. We also found that, in some cases, GEN was released with relatively high control, near-zero-order kinetics. The “burst release” of GEN has not been observed. Importantly, in vitro studies conducted on the skin cells have shown that the hydrogels do not show a cytotoxic effect on both fibroblasts and keratinocytes. In addition, the GEN-loaded hydrogels have a positive effect on the viability and proliferation of these cells. From a broader perspective, this study suggests that synthesized hydrogels may potentially be used in dermatology and cosmetology.

## Figures and Tables

**Table 1:** Synthesis of CL, *rac*-LA and PEG copolymers.

Sample	Molar Ratio ^a^	Yield [%]	*M_n_*^b^ [g/mol]	*Đ* ^b^	% mol CL or LA ^c^	% mol PEG ^c^
PLA-PEG-1	20:1	77	3200	1.69	79.7	20.3
PLA-PEG-2	25:1	72	3400	1.57	84.9	15.1
PLA-PEG-3	30:1	69	3500	1.62	88.3	11.7
PCL-PEG-1	30:1	88	3400	1.48	80.4	19.6
PCL-PEG-2	35:1	80	3500	1.43	84.3	15.7
PCL-PEG-3	40:1	76	3700	1.52	88.9	11.1

^a^*rac*-LA (or CL): PEG 400 molar ratio; ^b^ average molar mass and dispersity determined by the GPC method; ^c^ percent of mers molar content in the chain (determined by ^1^H NMR);Reaction conditions: temp. 80 °C, time—7days, medium—toluene, CALB (400 mg).

**Table 2:** Characterization of hydrogels.

Sample	PLA-PEG	PCL-PEG	MSR [%] ^a^	MSR [%] ^b^	MSR [%] ^c^
HPUCHT-1	PLA-PEG-1 (80:20)	-	375 ± 17	412 ± 18	421 ± 18
HPUCHT-2	PLA-PEG-2 (85:15)	-	311 ± 14	341 ± 15	348 ± 15
HPUCHT-3	PLA-PEG-3 (88:12)	-	284 ± 13	311 ± 13	326 ± 14
HPUCHT-4	-	PCL-PEG-1 (80:20)	297 ± 14	328 ± 15	333 ± 15
HPUCHT-5	-	PCL-PEG-2 (84:16)	258 ± 11	284 ± 13	287 ± 13
HPUCHT-6	-	PCL-PEG-3 (89:11)	211 ± 11	232 ± 11	243 ± 12

Reaction conditions: temperature—80 °C, time—3 h (the first step of the prepolymer process) and 4 h (the second step the of prepolymer process); HDI and PLA-PEG 400 (or PCL-PEG 400) molar ratio: 2.05:1 (in a −NCO/−OH); −NCO(prepolymer)/−OH(or −NH_2_) (CHT) molar ratio: 1.5:1; MSR—mass swelling ratio. ^a^—after 4 h; ^b^—after 24 h; ^c^—after 48 h.

**Table 3:** Analysis data of GEN release from the obtained hydrogels.

No.	Zero-Order Model	First-Order Model	Korsmeyer-Peppas Model		GEN Transport Mechanism
	R^2^	R^2^	R^2^	n	
HPUCHT-1	0.847	**0.915**	0.998	0.516	non-Fickian transport
HPUCHT-2	0.873	**0.940**	0.995	0.609	non-Fickian transport
HPUCHT-3	**0.921**	0.868	0.944	0.452	non-Fickian transport
HPUCHT-4	**0.923**	0.913	0.976	0.488	non-Fickian transport
HPUCHT-5	**0.925**	0.869	0.968	0.553	non-Fickian transport
HPUCHT-6	**0.941**	0.831	0.957	0.597	non-Fickian transport

**Table 4:** The results of the umu-test for the extracts tested.

	−S9 *		+S9 **	
	G ± SD	IR ± SD	G ± SD	IR ± SD
**MS H-1**	1.00 ± 0.04	0.94 ± 0.07	0.95 ± 0.02	0.96 ± 0.04
**MS H-2**	1.05 ± 0.05	0.82 ± 0.15	0.98 ± 0.05	0.85 ± 0.20
**MS H-3**	1.06 ± 0.08	0.81 ± 0.13	1.03 ± 0.06	0.96 ± 0.20
**MS H-4**	0.99 ± 0.04	0.94 ± 0.08	1.10 ± 0.24	0.85 ± 0.34
**MS H-5**	1.05 ± 0.08	0.84 ± 0.17	1.07 ± 0.14	0.88 ± 0.26
**MS H-6**	1.01 ± 0.03	0.93 ± 0.10	1.03 ± 0.15	0.90 ± 0.27
**Positive contol**	0.98 ± 0.01	7.77 ± 0.18	0.88 ± 0.02	4.52 ± 0.13
**Negative control**	1.01 ± 0.07	1.00 ± 0.13	1.00 ± 0.10	1.02 ± 0.25
**Solvent control**	1.02 ± 0.04	1.04 ± 0.14	0.74 ± 0.08	1.13 ± 0.20

* without metabolic activation; ** with metabolic activation.

## Data Availability

Data are contained within the manuscript.
